# Fatty Acid Signaling Mechanisms in Neural Cells: Fatty Acid Receptors

**DOI:** 10.3389/fncel.2019.00162

**Published:** 2019-04-24

**Authors:** Lisandro Jorge Falomir-Lockhart, Gian Franco Cavazzutti, Ezequiel Giménez, Andrés Martín Toscani

**Affiliations:** ^1^Instituto de Investigaciones Bioquímicas de La Plata (INIBIOLP), Centro Científico Tecnológico – La Plata, Consejo Nacional de Investigaciones Científicas y Técnicas (CONICET), La Plata, Argentina; ^2^Facultad de Ciencias Exactas, Universidad Nacional de La Plata (UNLP), La Plata, Argentina; ^3^Facultad de Ciencias Médicas, Universidad Nacional de La Plata (UNLP), La Plata, Argentina

**Keywords:** lipid sensing, neuronal differentiation and development, signal transduction, free fatty acid receptor, fatty acid binding protein, peroxisome proliferator activated receptor, docosahexaenoic acid, arachidonic acid

## Abstract

Fatty acids (FAs) are typically associated with structural and metabolic roles, as they can be stored as triglycerides, degraded by β-oxidation or used in phospholipids’ synthesis, the main components of biological membranes. It has been shown that these lipids exhibit also regulatory functions in different cell types. FAs can serve as secondary messengers, as well as modulators of enzymatic activities and substrates for cytokines synthesis. More recently, it has been documented a direct activity of free FAs as ligands of membrane, cytosolic, and nuclear receptors, and cumulative evidence has emerged, demonstrating its participation in a wide range of physiological and pathological conditions. It has been long known that the central nervous system is enriched with poly-unsaturated FAs, such as arachidonic (C20:4ω-6) or docosohexaenoic (C22:6ω-3) acids. These lipids participate in the regulation of membrane fluidity, axonal growth, development, memory, and inflammatory response. Furthermore, a whole family of low molecular weight compounds derived from FAs has also gained special attention as the natural ligands for cannabinoid receptors or key cytokines involved in inflammation, largely expanding the role of FAs as precursors of signaling molecules. Nutritional deficiencies, and alterations in lipid metabolism and lipid signaling have been associated with developmental and cognitive problems, as well as with neurodegenerative diseases. The molecular mechanism behind these effects still remains elusive. But in the last two decades, different families of proteins have been characterized as receptors mediating FAs signaling. This review focuses on different receptors sensing and transducing free FAs signals in neural cells: (1) membrane receptors of the family of G Protein Coupled Receptors known as Free Fatty Acid Receptors (FFARs); (2) cytosolic transport Fatty Acid-Binding Proteins (FABPs); and (3) transcription factors Peroxisome Proliferator-Activated Receptors (PPARs). We discuss how these proteins modulate and mediate direct regulatory functions of free FAs in neural cells. Finally, we briefly discuss the advantages of evaluating them as potential targets for drug design in order to manipulate lipid signaling. A thorough characterization of lipid receptors of the nervous system could provide a framework for a better understanding of their roles in neurophysiology and, potentially, help for the development of novel drugs against aging and neurodegenerative processes.

## Introduction

The central nervous system (CNS) is an intricate network of a variety of cell types, with a wide range of distinct properties and functions. Notoriously, lipids represent a larger proportion of mass than in most other tissues, second only after adipose tissue (Etschmaier et al., 2011). The main difference is that, whereas in adipocytes lipids are mainly stored as energy reserve in lipid droplets, in the CNS lipids fulfill multiple functions; such as forming large extensions of membranes necessary for crosstalk between neural cells. This is not limited only to the inner and plasma membranes of neurons, comprising axons, dendrites, and spines; astrocytes, oligodendrocytes, and microglia also have highly complex cellular shapes and, hence, extensive surface areas defined largely by lipids, and their functions in the healthy brain are integrated both physically and metabolically (von Bernhardi et al., 2016). Brain membranes are constituted by proteins, cholesterol (on average, 21.5% mol/mol lipids), sphingomyelin (1.9%), gangliosides (1.4%) and phospholipids (75.1%) (Scandroglio et al., 2008), where the lipids were typically constrained to structural or support functions for the first ones. Lipid distribution is not equivalent for all cell types or brain regions, and neurons are usually enriched in polyunsaturated glycerolipids and cholesterol (Scandroglio et al., 2008; Chan et al., 2012; Arai et al., 2015; Ingolfsson et al., 2017). These lipids are essential for many cellular processes in CNS. For example, the brain is the richest tissue in cholesterol with up to 20 mg/g of tissue, 10-time more than the rest, and it is mainly synthesized *in situ* (Dietschy and Turley, 2001, 2004). Therefore, its levels are independent from circulating cholesterol, highlighting its pivotal role in multiple brain functions such as signal transduction, synaptic transmission, and cell differentiation by modulation of lipid rafts organization and segregation of membrane proteins, as well as in several pathological conditions, either directly by its self or through its metabolism into neurosteroids and oxysterols (Reddy, 2010; Leoni and Caccia, 2011; Orth and Bellosta, 2012; Vance, 2012).

Lipid signaling has attracted increasing attention as different families of lipids have been shown to exhibit important regulatory functions through highly specific receptors (Fernandis and Wenk, 2007; Sunshine and Iruela-Arispe, 2017). Polyunsaturated fatty acids (PUFAs) are of particular relevance because they can also be transformed into much more potent derivatives, such as eicosanoids and docosanoids, from arachidonic (C20:4ω-6, ARA) and docosahexanoic (C22:6ω-3, DHA) acids, respectively (Serhan, 2014; Dennis and Norris, 2015). In the last decades, different families of proteins have been identified and characterized to mediate signaling processes triggered directly by lipids *per se*, including free FAs (Im, 2004; Kostenis, 2004; Wolfrum, 2007). The interest on how these proteins mediate FAs regulatory functions increased as they have been demonstrated to be targets and/or essential carriers for the therapeutic actions of certain drugs (Wolfrum et al., 2001; Landrier et al., 2004; Martin et al., 2013).

Fatty acids regulatory functions are most probably defined by the group of specific receptors expressed in a particular cell-type and, therefore, they are dynamically modulated throughout the cell life-span, the developmental stage, and its differentiation process. This review resumes part of the present understanding on FAs regulatory functions in pathophysiology of neural tissues and three families of proteins involved in these roles. Noteworthy, other proteins involved in lipid signaling with more promiscuous selectivity of ligands, such as FA Translocase (FAT/CD36) or Toll-like Receptor 4 (TLR4), were not included in this manuscript, and their roles could be consulted elsewhere (Pepino et al., 2014; Magnan et al., 2015; Rocha et al., 2016).

## Physiological Functions and Participation of Fatty Acids in Neuronal Pathologies

Fatty acids are typically considered as a source of energy through β-oxidation, and, as part of phospholipids, either as membrane building blocks or reservoir of second messengers and substrates for cytokines synthesis. They can also induce multiple cellular responses, ranging from cell motility and changes in cell morphology (Kamata et al., 2007; Brown et al., 2014), to regulation of gene expression (Kitajka et al., 2004), modulation of hormones secretion or their effects (Wang and Chan, 2015). But several distinctions must be made when referring to FAs in general, because saturated FAs (SFAs) have a clear different origin, metabolism, and functions compared to essential ω-3 and ω-6 PUFAs. Brain SFAs, such as palmitate (16:0, PA) or stearic (18:0, SA), are known to be generated *in situ* as much as being imported into the brain, whereas PUFAs are elongated and further unsaturated mainly in the liver and then transported through the bloodstream and imported into the neural tissue as non-esterified FAs (Edmond et al., 1998; Chen et al., 2008). Only a small fraction of PUFAs is actually synthesized locally from linoleic (C18:2ω-6) and α-linolenic (C18:3ω-3, ALA) acids; and, due to enzymatic restrictions and competition between ω-3 and ω-6 PUFAS for the same enzymes, less than 5% of total ALA assimilated from diet can be transformed into DHA (Dyall and Michael-Titus, 2008). Both, endogenous and blood-derived PUFAs, accumulate preferentially in neurons as part of phospholipids.

Neurogenesis includes three contiguous phases, namely proliferation, migration and differentiation, and maturation and integration of the precursor cells; with PUFAs having inherence in all of these stages (Chalon, 2006). Therefore, PUFAs are critical for pre- and post-natal brain development, as well as in adulthood or during natural aging (Uauy and Dangour, 2006; Rombaldi Bernardi et al., 2012). For example, ALA maternal restriction during gestation and lactation impairs hippocampal neuronal differentiation, thus compromising neuronal maturation and related brain functions, such as learning and memory (Bhatia et al., 2011; Niculescu et al., 2011). DHA is the most abundant PUFA and accumulates in the immature brain during perinatal life all through the grey matter expansion; and it can reach over 10% of total FAs in human adult brains (McNamara and Carlson, 2006). Actually, preterm-born adolescents, who skipped the fetal DHA accumulation in the CNS during the last weeks of gestation and usually also lactation during the early life, exhibit deficits in cognitive functions associated to attention, including increased risk for attention-deficit/hyperactivity disorder (ADHD) and schizophrenia (McNamara and Carlson, 2006). That is why dietary deficiencies commonly stimulate a more tightened retention of essential FAs in the brain.

Docosahexanoic acid is particularly important for proper neuronal development in cerebral cortex, retina, and hippocampus, where it promotes neurogenesis and neuronal differentiation (Cao et al., 2009; Gharami et al., 2015). It also boosts synaptogenesis by promoting neurite outgrowth and synapsis formation, accompanied by an increase in the expression of neuronal and synaptic proteins. Particularly, in rat neuronal stem cells, DHA stimulates neuronal differentiation by two mechanisms: (1) a decrease in expression levels of basic Helix-loop-Helix transcription factors (NeuroD, Mash1, Hes1, etc.); and (2) an extension of the expression of cyclin-dependent kinase inhibitor p27 (kip1). This results in an increase in the number of positive cells for the neuronal markers TUBB3 and MAP2 and in a reduction of the percentage of cells in S-phase, suggesting an exit from the cell cycle (Katakura et al., 2009). In aged mice, DHA also prevents neuroinflammation and apoptosis, whereas improving memory (Labrousse et al., 2012). DHA can prevent many of the lipopolysaccharide (LPS) deleterious effects on both neurons and microglia, including loss of dendritic spines or production of nitric oxide as biomarkers of neuroinflammation (Chang et al., 2015). Actually, multiple animal and *in vitro* models of neuroinflammation consistently show a marked anti-inflammatory effect of DHA and other ω-3 PUFAs, presumably by reducing the production of proinflammatory cytokines and/or promoting the secretion of anti-inflammatory cytokines (reviewed in Orr et al., 2013). PUFAs can also directly modify neurotransmitter production, accumulation, release, and re-uptake. Dopamine’s content, storage in presynaptic vesicles and tyramine-stimulated release, for example, significantly decrease in rat frontal cortex after chronic ω-3 PUFAs deficiency (Delion et al., 1996; Zimmer et al., 1998, 2000). Contrarily, supplementation with ω-3 PUFAs increases dopamine levels in the same area (Chalon et al., 1998).

Fatty acids have been implicated in neuropathological conditions, including neurodegenerative diseases, mental disorders, stroke, and trauma. Severe dietary restriction of essential FAs usually correlates with anxiety-like behavior and deficit in cognitive functions, including memory and learning. However, imbalance between ω-3 and ω-6 PUFAs during gestation also leads to alterations in brain development. In adults, an imbalance in the PUFAs ratio in brain membranes is believed to be a risk factor active during the pathogenesis of neurological and psychiatric disorders (Chalon, 2006). For example, an increase in ARA is known to promote α-Synuclein aggregation. However, it is not clear which is the most relevant cause affecting secretion of dopamine and serotonin, the greater availability of ARA, the deficiency of DHA or the imbalance between them (Chalon, 2006). During normal aging, there is a common cognitive decline and an increasing risk of dementia. High ω-3 PUFAs intake slows down this decline (Johnson and Schaefer, 2006; van Gelder et al., 2007), suggesting that they may have neuroprotective action in the aging brain and even therapeutic potential. Aging is also characterized by increased oxidative stress and neuroinflammation together with altered energy metabolism (reviewed in Prolla and Mattson, 2001). Beside changes in FA composition that favor monounsaturated FAs (MUFAs) over ω-3 and ω-6 PUFAs with time in rodents, the aging brain is also prone to lipid oxidation due to its large content of PUFAs (Favrelere et al., 2000). In humans, the decrease of PUFAs, though subtler during normal aging, is quite evident in neurodegenerative pathologies, including Alzheimer Disease (AD), Parkinson’s Disease (PD), Schizophrenia and depression (reviewed in Hussain et al., 2013). Nevertheless, PUFAs are known to promote α-Synuclein pathological aggregation (Yakunin et al., 2012). On the other hand, the anterior cingulate cortex of depressive patients shows reduced quantities of SFAs and PUFAs (Conklin et al., 2010). The deficiency of ω-3 PUFAs also impairs the normal signaling of endocannabinoid in prelimbic prefrontal cortex and accumbens, leading to abnormal emotional behavior (Lafourcade et al., 2011). The reduction in total PUFAs in erythrocyte’s membranes of patients with early onset schizophrenia correlates with the degree of demyelination in brain white matter (Peters et al., 2009). Finally, PUFAs also serve as anti-depressants and anti-convulsants, confer protection against traumatic insults, and enhance repairing processes. For example, numerous animal models of epilepsy support the anticonvulsant properties of PUFAs, like the greater resistance to pentylenetetrazol-induced seizures in ω-3 PUFAs fed rats (Taha et al., 2009).

Arachidonic acid is particularly enriched in phosphatidylinositol (PI), whereas DHA is a major component of brain phosphatidylethanolamine (PE) and phosphatidylserine (PS). Both PUFAs are highly enriched in the phospholipids of the synaptic plasma membrane and synaptic vesicles (Glomset, 2006). ARA is also particularly rich in membranes of leukocytes, presumably because it serves as a precursor of a myriad of cytokines (Innes and Calder, 2018). PUFAs are almost exclusively found in position 2 of the glycerol moiety within any phospholipid and, therefore, their release is catalyzed by phospholipases A_2_ (PLA_2_). Noteworthy, ARA and eicosapentaenoic acid (20:5ω-3, EPA) are released preferentially by cytoplasmic PLA_2_ (cPLA_2_), whereas DHA is released by Ca^+2^-independent PLA_2_ (iPLA_2_) (Dyall and Michael-Titus, 2008). These three PUFAs can be converted into more potent cytokines than the free FAs, including prostaglandins, leukotrienes, thromboxanes, protectins and resolvins, and their effects differ from one other (Calder, 2015; Dyall, 2017).

Neuroinflammation is a common denominator for several neuropathologies. SFAs are known to promote the inflammatory phenotype of microglia, stimulating the secretion of TNF-α and IL-6 through TLR4. Furthermore, PA exposure of astrocytes leads to Caspase-3 activation and alters Bax/Bcl-2 ratio, both effects promoting apoptosis (Gupta et al., 2012). On the contrary, ω-3 PUFAs usually have a marked antiinflammatory effect by blocking microglia activation and stimulating the secretion of neurotrophic factors. However, some deleterious effects have also been reported, such as the worsening of neuritic injury and astrocytosis in PD-mice model (Hussain et al., 2013). ARA is the precursor for potent proinflammatory eicosanoids. On the other hand, compounds derived from DHA and EPA, known as resolvins and protectins, show strong anti-inflammatory effects and mediate the end of an ongoing inflammatory response (Hussain et al., 2013). These compounds are just an example of those derived from FAs that have important roles in brain pathophysiology. A special mention should be given to endocannabinoids, such as arachidonoylethanolamine (also known as anandamide), arachidonylglycerol or oleoylethanolamine, just to name a few (Freitas et al., 2017). Nevertheless, the role of free FAs has a renewed interest since the identification of multiple receptors that can couple their signaling to diverse cellular responses. The next section focusses on three families of proteins that recognize FAs and could be modulating FAs regulatory functions.

## Receptors for Fatty Acids Signaling

Originally, lipid signaling was limited to steroid hormones and its binding to cytosolic receptors, or to cytokines derived from ARA (leukotrienes and thromboxanes) that bind to specific membrane receptors. As of today, three families of proteins have been identified to be able to sense the presence and type of FAs whether in the extracellular medium, the cytosol or the nuclear matrix. At the plasma membrane, FAs can activate G Protein-coupled receptors known as Free Fatty Acid Receptors (FFARs) (Hara et al., 2013; Offermanns, 2013); while in the cytosol they can be taken by Fatty Acid Binding Proteins (FABPs) and targeted to specific subcellular structures or metabolic pathways (Veerkamp and Zimmerman, 2001; Storch and Corsico, 2008). Finally, nuclear receptors Peroxisome Proliferator-Activated Receptors (PPARs) mediate FAs regulatory functions in the nucleus (Zolezzi et al., 2017). The specific spatio-temporal pattern of expression and the co-expression of more than one isoform from each family of proteins in a single cell suggest a platform for sensing and modulating the cellular response to the bioavailability of the different FAs, for example, adapting the cell to developmental or functional requirements. Therefore, the regulatory and signaling roles of free FAs are gaining importance in physiological and pathological processes as these receptors are better characterized.

We describe bellow some of the known characteristics of each family of FAs receptors selected for this review, including expression patterns, structural features, and specific functions in the CNS. Noteworthy, when the source of expression data is not specified, it was taken from The Human Protein Atlas website^1^ (Uhlen et al., 2015), based on Genotype-Tissue Expression (GTEx) project (The GTEx Consortium, 2013), or from the Allen Mouse Brain Atlas^2^ (Lein et al., 2007).

### Plasma Membrane Receptors

A large number of putative genes coding for GPCRs was identified based on genome sequences, and their deorphanization is still in progress (Civelli, 2005; Diaz et al., 2018; Laschet et al., 2018). Particularly, a cluster of 4 sequences (*GPR40*, *GPR41*, *GPR42* and *GPR43*) was identified in chromosome 19 (Sawzdargo et al., 1997) and FAs were proved to work as their specific endogenous ligands (Briscoe et al., 2003; Brown et al., 2003), dubbing this subfamily of GPCRs as FFARs (Offermanns, 2013). Later on, two other receptors activated by FAs were described, GPR84 and GPR120, located in chromosome 12 and 10, respectively; as well as many others activated by diverse lipids derived from FAs, including lysophosphatidic acid (termed LPARs), endocannabinoids (CBs) and hydroxycarboxylic acids (HCAs) (Offermanns, 2013; Audet and Stevens, 2019).

**FIGURE 1:**
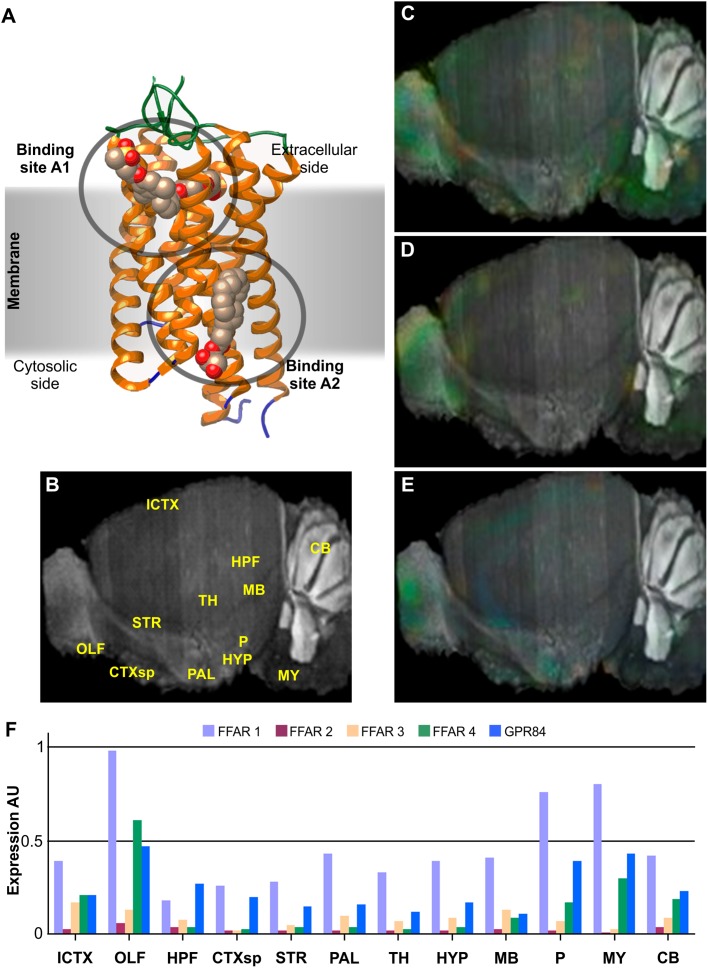
Structure and brain expression patterns of FFARs. **(A)** Cartoon based on FFAR1 crystal structure (PDB-ID: 4PHU) (Srivastava et al., 2014), highlighting membrane orientation and two ligand binding sites in opposite sides of the membrane. **(B)** Central brain section of 8-weeks, male mouse (C57BL/6J) indicating reference regions. **(C–E)** Brain left hemisphere sagittal projection showing expression of FFAR1, FFAR4 and GPR84, respectively, based on *in situ* hybridization (ISH) data. FFAR2 and FFAR3 are not shown due to its lower expression levels. **(F)** Quantification of relative expression from ISH data for all FFARs isoforms. AU: Arbitrary units; ICTX: Isocortex; OLF: Olfactory areas; HPF: Hyppocampal formation; CTXsp: Cortical subplate; STR: Striatum; PAL: Pallidum; TH: Thalamus; HYP: Hypothalamus; MB: Midbrain; P: Pons; MY: Medulla; and CB: Cerebellum. Image credit for panels **C–E**: Allen Institute © 2007 Allen Institute for Brain Science. Allen Mouse Brain Atlas. Available from: http://mouse.brain-map.org/search/. Panels **B** and **F** were constructed from data available through Allen Mouse Brain Atlas website.

Within the superfamily of GPCRs, FFARs belong to the largest subfamily of Class A/1 (rhodopsin-like) receptors, constituted by a motif of 7 transmembrane segments (TMs) and at least one longer cytosolic domain that serves as binding site for signaling machinery assembly (Dorsam and Gutkind, 2007; Heldin et al., 2016). The ligand recognition site is defined within the transmembrane helix bundle (Figure 1A). The original cluster of FFARs was deorphanized by heterologous expression and ligand screening through monitoring cytosolic Ca^+2^ levels (Briscoe et al., 2003). FAs-triggered Ca^+2^ release from the endoplasmic reticulum is PI3K-dependent. However, some of these receptors can also transduce FAs signal through inhibition of cAMP synthesis, CREB, Akt/PKB, and/or Erk phosphorylation, as well as by β-Arrestin recruitment (Kimura et al., 2011; Zamarbide et al., 2014). Each FFAR shows specific patterns of expression and distinctive ligand selectivity. Their functions have been better studied in peripheral tissues, including the promotion of hormone secretion by the pancreas, lipid taste sensing and the activation of immune cells as a preliminary response toward a putative growing infection. Their participation in the CNS had less attention, although recent results suggest that some isoforms could participate in brain development, neuronal differentiation (Ma et al., 2010) and could be potential drug targets against several neuropathies, such as neuropathic pain and epilepsy (Yoshimura et al., 2015; Yang et al., 2018).

Each isoform presents distinct body expression profiles and different affinity for FAs (Brown et al., 2005; Hara et al., 2013; Offermanns, 2013). There are two FFARs with prominent levels of expression in neural tissues: FFAR1 (also known as GPR40) and FFAR4 (GPR120 or O3FAR). Nevertheless, all isoforms have been detected, though weakly, in some restricted brain region. We review bellow some of the most recent findings about this novel family of GPCRs activated by FAs.

#### FFAR1 (GPR40)

The genes that code for FFAR1, FFAR2 and FFAR3, originally named *GPR40*, *GPR43* and *GPR41*, respectively, were identified in 1997 as a group of tandemly encoded genes located on human chromosome 19q13.1. FFAR1 is a 300 amino acids membrane protein of 31.45 kDa. As the product of the single exon gene *GPR40*, it is most abundantly expressed in pancreas and brain of primates (Briscoe et al., 2003; Brown et al., 2005). Other tissues with significant expression include small intestine, spleen, testis, ovaries, and Fallopian tubes. According to GTEx data, within the CNS, higher expression levels of FFAR1 mRNA are found in hippocampus, caudate nucleus, hypothalamus, and cerebral cortex, presumably mainly in neurons but also in glial cells. On the other hand, qPCR targeted experiments showed a wider expression of FFAR1 in neuronal tissues, with higher expression levels in medulla oblongata, *substantia nigra* and spinal cord, followed by putamen, locus cereleus, globus palidus, and amygdala (Briscoe et al., 2003), while western blot analysis showed higher expression in pons, dentate gyrus, pituitary gland, *substantia nigra* and spinal cord, followed by subgranular and subventricular zones, CA1, medulla oblongata, and cerebral cortex, with minor expression in cerebellum (Ma et al., 2007). *In situ* hybridization (ISH) of mice brain sagittal sections indicated discrete preferential FFAR1 expression in olfactory bulb, pons, and medulla (Figure 1C,F), while coronal sections highlighted hippocampus and cerebral cortex (Zamarbide et al., 2014). Its levels are weaker than in primates and, hence, it has been suggested that is not essential. Actually, FFAR1-KO mice manifested no evident alteration of social or motor behavior, although they showed a reduction anxiety-like responses (Mancini et al., 2015). An increase in noradrenaline was also observed in FFAR1-KO mice brain regions were the expression of the receptor was shown to be higher, suggesting a role in anxiety and depression symptoms.

This receptor is the only FFAR with solved crystal structures (PDB-ID: 5KW2, 4PHU, 5TZR and 5TZY), as different fusions to Lysozyme, and in complex with different partial and full synthetic agonists, such as TAK-875, MK-8666 or the novel compound (3∼{S})-3-cyclopropyl-3-[2-[1-[2-[2,2-dimethylpropyl-(6-methylpyridin-2-yl) carbamoyl]-5-methoxy-phenyl]piperidin-4-yl]-1-benzofuran-6-yl] propanoic acid (Srivastava et al., 2014; Lu et al., 2017; Ho et al., 2018). Noteworthy, the different structures identified two putative binding sites in opposite sides of the transmembrane region, A1 in the extracellular side and A2 in the cytosolic one (Figure 1A). Only the occupancy of the latter by synthetic or natural ligands promotes full agonistic response and structure of intracellular loop IC2 located between transmembrane regions TM3 and TM4, which is believed to be necessary for G-protein interaction. The extracellular loop located between TM4 and TM5 is stabilized by a disulfide bond formed between Cys^79^ and Cys^170^. Residues participating in ligand recognition have been mapped to include Arg^183^, Asn^244^ and Arg^258^ that work as anchors for the carboxylic head of the FAs. Residues Tyr^12^, Tyr^91^, His^137^, and Leu^186^ were also identified as relevant for receptor activation; while His^86^, Tyr^91^, His^137^ and the anchoring positions seem to be critical for the binding and activation by the synthetic agonist GW9508 (Sum et al., 2007; Tikhonova et al., 2007).

FFAR1 has approximately the same high affinity (EC_50_ 5-60 μM) for medium and long chain saturated FAs ranging from C6 (caproic acid) to C23 (tricosanoic acid), with FAs between C10 to C18 being the preferred endogenous agonists; but it is not active in the presence of acetate, propionate, butyrate, or pentanoate (valerate). It also shows the same range of high affinities for both MUFAs and PUFAs, with ARA (C20:4ω-6), EPA (C22:5ω-3), and DHA (22:6ω-3) exhibiting the highest potencies (Briscoe et al., 2003; Offermanns, 2013). Noteworthy, retinoids can also activate FFAR1 with similar affinities, and, particularly, all-trans-retinoic acid, a well-known factor required for neuronal differentiation (Yu et al., 2012; Janesick et al., 2015), shows one of the highest affinities, EC_50_ < 5 μM (Briscoe et al., 2003).

In pancreas, its activation stimulates insulin secretion from β-cells (Itoh et al., 2003), involving signaling by both Gα_q/11_ subunit and β-Arrestin2 (Mancini et al., 2015), what does have an impact on systemic metabolism control. Diverse functions have been proposed for FFAR1 in the CNS or the peripheral sensory system. Cartoni et al. (2010) described the expression of FFAR1 in type I cells of the taste buds, along with FFAR4 in type II cells, both participating in the oral perception of fats. FFAR1 has also been associated to neurogenesis and linked to cognitive functions such as memory, space orientation and learning (Ma et al., 2008; Yamashima, 2008; Boneva et al., 2011; Zamarbide et al., 2014). Although these effects are probably mediated mainly by phosphorylation of CREB, its signaling pathway is still not well understood. Since Gα_s_ proteins do not seem to be activated by FFAR1, an alternative phosphorylation of CERB via Erk has been proposed (Yamashima, 2012). Differentiation and maturation of cultures of rat neuronal stem cells overexpressing FFAR1 can be stimulated by DHA, promoting neurite outgrowth and branching, even at concentrations as low as 1,5 μM (Ma et al., 2010). FFAR1 has also been proved to mediate the antinociceptive activities of DHA via induction of secretion of μ and δ opioid peptides, but not κ opioid peptides (Nakamoto et al., 2012). In the hypothalamus FFAR1 is expressed in NPY and POMC expressing neurons linked to satiety and control of food intake, and its activation by its synthetic agonist TUG905 increase POMC (anorexigenic precursor) secretion that leads to body weight loss (Dragano et al., 2017).

High fat diet in mice and PA-albumin complex in neuroblastome derived cells in culture were shown to stimulate synthesis of amyloid precursor protein (APP) and β-site APP cleaving enzyme 1 (BACE1), promoting the release of Aβ peptides in hippocampus and brain cortex, similarly to Akt pathways activation by GW9508 agonist of FFAR1 (Kim et al., 2017). This effect can be blocked in SK-N-MC cells *in vitro* if FFAR1 is pharmacologically inhibited with GW1100, knocked down by siRNA or delocalized from lipid rafts when membranes are depleted from cholesterol with methyl-β-cyclodextrin. However, GW9508 activation of FFAR1 in a mouse model of AD, based on Aβ intracerebroventricular (icv) injection, leads to significant improvement in cognitive and behavioral tests, what seems to be mediated by CREB phosphorylation and the concomitant increase in expression of NGF and BDNF neurotrophic factors. These effects were not observed when the inhibitor GW1100 was co-administered with the synthetic agonist (Khan et al., 2016). As these opposing effects originate from divergent signaling pathways from a single receptor, exploitation of FFAR1 synthetic agonists or inhibitors for treating neuropathologies may be a task worth pursuing. But first, it is imperative to fully characterize the signal transduction mechanisms active in the CNS, as well as to develop specific drugs that can selectively activate only those promoting neuroprotection, neuronal differentiation, and maturation. During the last decades, an increasing amount of evidence was collected linking the etiology and pathology of AD with Type-2 Diabetes Mellitus (Benedict and Grillo, 2018; Ferreira et al., 2018; Chornenkyy et al., 2019). Considering the wide range of synthetic agonist of FFAR1 being developed to treat Diabetes, it became an interesting possibility to evaluate their repurposing for treating AD as well (Li et al., 2016; Chen et al., 2019). For example, two different mouse models of diabesity (diabetes and obesity), based on high fat diet or db/db mice, present a decrease in FFAR1 and BDNF expression in the hippocampus and brain cortex, that can be reverted by chronic administration of DHA or GW9608 (Sona et al., 2018). Since it can be inhibited by Erk and MAPK inhibitors, BDNF expression depends on FFAR1-pErk pathway.

#### FFAR2 (GPR43)

Although the gene structure of GPR43 is not yet fully defined, FFAR2 was described to be encoded as a 330 amino acids protein (37.14 kDa) in a single exon. Its structure is predicted to have a topology very similar to FFAR1, where the extra 30 amino acids would correspond mainly to a longer C-terminal tail. However, FFAR2 preferential endogenous ligands are limited to acetate, propionate, butyrate, and pentanoate (with EC_50_ somewhat an order of magnitude larger than those for FFAR1), and this receptor does not respond to ligands with chain length above C6 (Brown et al., 2003; Offermanns, 2013). Its activation has been describe to be coupled both to pertussis toxin sensitive (Gα_i_) and insensitive (Gα_q_) proteins (Brown et al., 2003; Stoddart et al., 2008). In the first pathway, FFAR2 activation leads to a decrease in cAMP production by adenylate cyclase (AC) and, therefore, to a reduction in protein kinase cAMP-activated (PKA) activity. On the other hand, Gα_q_ activates phospholipase C (PLC) that cleaves phosphatidylinositol bisphosphates (PIP_2_) into inositol-triphosphate (IP_3_) and diacylglycerol. IP_3_ binds to IP_3_R receptor in the endoplasmic reticulum and induces the internal release of Ca^+2^ to the cytosol, which then activates Ca^+2^-dependent protein kinase C (PKC). DAG attracts certain PKC isoforms to the plasma membrane and helps to direct its activity to a subset of potential target substrates.

FFAR2 is most abundantly expressed in spleen, bone marrow, and lung, followed by adipose tissue, breast, and all the digestive tract. Its expression is mainly consistent with a leukocyte markers expression pattern, with higher presence in macrophages and leukocyte germ line (Brown et al., 2003). The low level, but widespread FFAR2 expression may be due to its presence in immune cells, such as infiltrating neutrophils and macrophages. Its expression in CNS is rather low compared to other FFARs (Figure 1F) and limited to glia and neurons of the caudate, but FFAR2 can also be detected in cortical neurons and pituitary gland. However, the participation of FFAR2 in neuronal processes still needs further analysis.

#### FFAR3 (GPR41)

FFAR3 is the product of the gene *GPR41*, only 6.62 kb downstream of *GPR40* promoter, but it lacks a formal promoter of its own. Although 3 sites of transcription initiation can be predicted in putative exons upstream *GPR41*, none of them could be confirmed. Contrarily, *GPR41* may be expressed as the result of the skipping of a termination sequence immediately downstream FFAR1 stop codon. Bahar Halpern et al. (2012) demonstrated that *GPR40* and *GPR41* are transcribed as a single bicistronic mRNA thanks to the action of an H2R enhancer, and that a tissue-specific internal ribosome entry site controls the translation of *GPR41* into FFAR3 in pancreatic β-cells. FFAR3 is also coded in a single exon, as a 346 amino acids protein (38.,65 kDa). *GPR40*-common origin is inferred from the relatively high sequence similarity/identity (31/34%), compared to other GPCRs. Like FFAR2, its structure is believed to be similar to FFAR1, with the extra 46 amino acids mainly corresponding to an extension of the cytosolic C-terminal tail; and its activation is mainly due to the binding of the same short chain FAs (Brown et al., 2003). The difference is that pentanoate is more potent than acetate for FFAR3, while it is quite the opposite for FFAR2. FFAR3 activation has been described to be coupled only to Gαi subunits. Its activation by β-hydroxy-butirate negatively regulates the activity of Cav2.2 N-type Ca^+2^ channel (Won et al., 2013; Colina et al., 2018).

High-throughput RNAseq screening analysis of FFAR3 expression indicated a widespread, but weak pattern, with higher levels observed in adipose tissue, breast, spleen, and digestive tract. Neural expression is scarce (Figure 1F) and only very weakly detected in the pituitary gland in GTEx (The GTEx Consortium, 2013). A GPR41-mRFP transgenic mice model published by Nohr et al. (2013) showed FFAR3 expression in the digestive tract intimately associated to the enteroendocrine system, mainly in enteric neurons of the submucosal and myenteric ganglia, and in several of the postganglionic sympathetic and sensory neurons, both in autonomic and somatic peripheral nervous system (PNS). But this model showed no expression in the brain or spinal cord. FFAR3 enteroendocrine expression links signaling of short FAs from the microbiota with enteric hormone secretion (Samuel et al., 2008). Its activation by short chain FAs and β-hydroxy-butirate in sympathetic neurons also inhibited Ca_V_2.2 currents, linking the functions of the PNS to the metabolic state (Won et al., 2013).

#### FFAR4 (GPR120)

The gene that codes for FFAR4, originally called *GPR120*, was described in 2003 to have four exons that are located on human chromosome 10q23.33 (Fredriksson et al., 2003). It is expressed, at least, as two variants generated by alternative splicing that leads to exon 3 skipping, *GPR120S* (coding for a 361 amino acids peptide, 40,49 kDa) and *GPR120L* (377 amino acids, 42,24 kDa). Predicted transmembrane regions leave N- and C-terminal segments longer than in previous FFARs, but the most notorious difference is the cytoplasmic loop between TM5 and TM6, where the *GPR120S* lacks 16 amino acids (from 233 to 248) compared to the longer variant *GPR120L*. Activation of FFAR4 signals through Gα_q/11_.

Highest expression of FFAR4 includes lower digestive tract, brain, adipose tissue, lung, testis, breast, and adrenal glands. Within the CNS, FFAR4 is found mainly in glial and neuronal cells of hippocampus, cerebral cortex, hypothalamus, and cerebellum (only granular layer and Purkinje cells), with a particularly high expression in pituitary gland (The GTEx Consortium, 2013). ISH of mice brain sections show FFAR4 preferential expression in medulla, pons and olfactory bulb, followed by hypothalamus and cerebellum (Figure 1D,F).

FFAR4 natural ligands include saturated FAs of C14, C16 and C18, with affinities around EC_50_ = 30, 52 and 18 μM, respectively. When overexpressed in HEK293 cells, both agonist-stimulated GPR120S and GPR120L receptors recruit β-Arrestin2 and undergo internalization, but the longer variant shows much less Ca^+2^ mobilization and average cellular response (Watson et al., 2012). Two Arg residues on the outer edge of TM2 (Arg^99^) and TM4 (Arg^178^) have been mapped to be responsible for FAs binding (Watson et al., 2012), unlike the other FFARs members that have two conserved Arg residues on the outer edge of TM5 and TM7 (Sum et al., 2007; Stoddart et al., 2008), showing once more the evolutionary divergence of FFAR4 from the rest of the GPCRs activated by FAs. Other residues involved in ligand binding and activation of the receptor included four aromatic residues Phe^115^, Phe^211^, Trp^277^, and Phe^304^ that, when mutated, essentially eliminated responsiveness to agonists (Hudson et al., 2014).

FFAR4 has been more thoroughly studied in adipocytes and intestine where it promotes insulin sensitizing and anti-inflammatory effects, respectively (Song et al., 2017). In enteroendocrine cells it helps controlling the secretion of hormones, promoting glucagon-like peptide 1 release but inhibiting ghrelin secretion (Hirasawa et al., 2005; Engelstoft et al., 2013); whereas, in adipocytes, oleic acid stimulates lipid droplet formation by activation of this receptor, which signals through Gα_q_, PI3K-Akt, and PLC pathways (Rohwedder et al., 2014; Villegas-Comonfort et al., 2017). However, it has a greater affinity for PUFAs, particularly those from the ω-3 series. In Caco-2 cells, EPA, DHA, and ARA elicit the same signaling events through FFAR4, but with different kinetics and efficiency (Mobraten et al., 2013). Basal and heterologous phosphorylation of Thr^347^, Ser^350^, and Ser^357^ in the C-terminal tail (GPR120S) mediates receptor internalization (Burns et al., 2014; Sanchez-Reyes et al., 2014). Hypothalamic expression of FFAR4 corresponds to microglia and its activation ameliorates neuroinflammatory response by decreasing the production of proinflammatory cytokines (TNFα and IL-1β) and promoting those with anti-inflammatory action (IL-6 and IL-10) (Dragano et al., 2017).

#### GPR84

This isoform was identified, through an EST library, in B cells encoded in a single exon (exon2) (Wittenberger et al., 2001), but it was the last one to be deorphanized (Wang et al., 2006). Therefore, GPR84 is the least studied isoform of the FFARs and has not yet been assigned with the “FFAR5” name. Its expression was confirmed by Northern-blot in brain, colon, thymus, spleen, kidney, liver, intestine, placenta, lung, leukocytes, heart, and muscle. Regarding CNS expression, it is most abundantly found in medulla and spinal cord, but also significantly in amygdala, *substantia nigra*, thalamus, and *corpus callosum*, whereas only weakly detected in other brain regions such as the cerebellum (Wittenberger et al., 2001) (Figure 1E,F). GPR84 is activated only by medium length acyl chain FAs (C9 to C14) and does not recognize longer or shorter chain carboxylic acids, promoting Ca^+2^ mobilization and inhibiting cAMP production, mainly through the activation of Gα_i/o_ (Wang et al., 2006). Its expression is augmented in monocytes by incubation with LPS, and its activation by medium chain FAs exacerbates the production of proinflammatory cytokines, highlighting GPR84 role in immune responses (Wang et al., 2006; Muller et al., 2017). Alternatively to endogenous ligands, GPR84 can also be activated by hidroxylated medium chain FAs and synthetic drugs like ZQ16 (2-(hexylthio)pyrimidine-4,6-diol), diindolylmethane or 6-n-octylaminouracil (Suzuki et al., 2013; Nikaido et al., 2015; Zhang et al., 2016).

When activated, GPR84 expressed in mouse primary cultures of microglia stimulates membrane ruffling, modifies its morphology, and promotes motility, but it does not promote an inflammatory response, including the secretion proinflammatory cytokines (Wei et al., 2017). Different FAs are known to be released after diverse brain injuries, from traumatic lesions to neurodegenerative diseases and neuroinflammatory conditions, and they may function as chemo-attractants for microglia, especially short and middle chain FAs that exhibit higher solubility and that can be recognized by FFAR2, FFAR3, and GPR84. This suggests that GPR84 could be a valid therapeutic target in microglia-associated diseases, such as AD or Multiple Sclerosis.

### Citosolic Receptors

**FIGURE 2:**
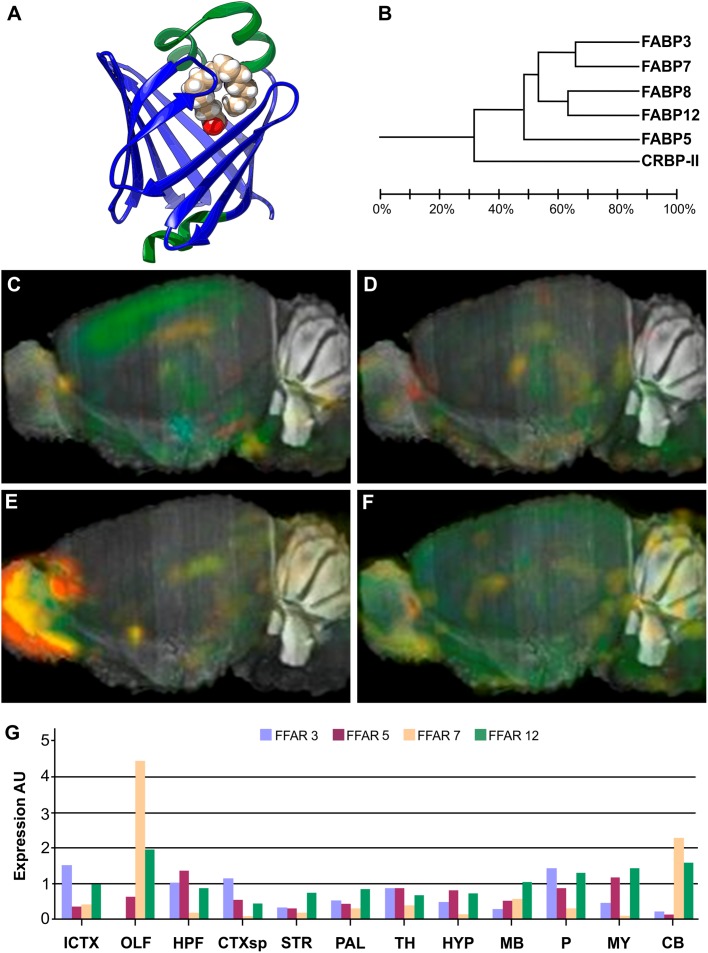
Structure and brain expression patterns of FABPs. **(A)** Cartoon based on crystal structure of human FABP3 complex with palmitic acid (PDB-ID: 6AQ1) (Yan et al., 2018). The β-barrel, array of 10 antiparallel β-strands, is shown in blue, and the helix-tur-helix motif cap is shown in green, with the palmitic acid (in filled-balls model) adopting a U-shaped conformation inside. **(B)** Dendrogram showing relationship between identity of sequence between FABPs expressed in neural tissues. Also belonging to the same folding, CRBP-II was included as and outlier. **(C–F)** Brain left hemisphere sagittal projection showing expression of FABP3, FABP5, FABP7 and FABP12, respectively, based on ISH data. FABP8 expression in mice brain is not significant. **(G)** Quantification of relative brain expression from ISH data for neural FABPs isoforms. AU: Arbitrary units. For brain regions references see legend of Figure 1. Image credit for panels **C**–**F**: Allen Institute. © 2007 Allen Institute for Brain Science. Allen Mouse Brain Atlas. Available from: http://mouse.brain-map.org/search/. Panel **G** was constructed from data available through Allen Mouse Brain Atlas website.

Due to their low solubility, free FAs need to be bound to proteins in order to diffuse through the aqueous medium of the cytosol. Since the 1970s, the proteins known as FABPs have been studied and proved to help overcome this problem. They are small (15 kDa) intracellular soluble proteins that reversibly bind FAs and other hydrophobic ligands, trafficking them to different intracellular compartments, such as mitochondria, peroxisome, endoplasmic reticulum or the nucleus. Even with as low as 30% identity of amino acids sequence, they share a highly conserved structure (Figure 2A,B), consisting in a barrel of 10 β-strands enclosing the binding cavity, and a helix-turn-helix motif capping this barrel. The latter regulates both ligand entry and exit, as well as origin and destiny points of traffic (Banaszak et al., 1994; Thompson et al., 1999; Storch and Corsico, 2008; Storch and Thumser, 2010). These proteins have been extensively characterized *in vitro*, including its three-dimensional structures by NMR and X-ray Crystallography, with and without any cargo, natural or synthetic (Sacchettini et al., 1987; Xu et al., 1991; Ory et al., 1997; Balendiran et al., 2000; Hanhoff et al., 2002; Rademacher et al., 2002; Reese and Banaszak, 2004). They can uptake FAs from model phospholipid vesicles and transfer its cargo to other membranes, according to the properties of each isoform (Herr et al., 1996; Storch and Thumser, 2000; Veerkamp and Zimmerman, 2001; Liou et al., 2002; Storch et al., 2002; Falomir-Lockhart et al., 2006). It has been demonstrated that some isoforms interact better with membranes in the apo-form, presumably looking for ligand uptake; whereas others are dominated mainly by electrostatic interaction between the holo-protein and the phospholipid’s headgroups (Falomir-Lockhart et al., 2006, 2011).

FABPs were originally named after the tissue where each isoform was firstly identified, and intestinal enterocytes were the exception that expressed large quantities of two isoforms, FABP1 and FABP2. Nowadays, the numbered nomenclature is preferred over tissue-related names in order to avoid misinterpretations derived from the fact that most cell types can express more than one member of the FABP family, and their level of expression responds to metabolic conditions, external stimuli, or development stages. Therefore, most FABPs show distinctive expression patterns in different organs and cell types. No isoform shows a high degree of selectivity for a particular FA, although it is usually found a higher affinity for saturated compared to unsaturated FAs (Richieri et al., 2000). The affinity for FAs of each isoform depends on chain length and number of double bonds, but isoforms FABP1 and FABP5 show significant affinity for a wider range of hydrophobic ligands. A total of 9 FABP isoforms with high affinity for long chain FAs (between C12 and C22) are described in mammals, plus 4 more members that show specificity for retinoid ligands (CRBP-I, CRBP-II, CRABP1 and CRABP2) and one extra for bile acids (FABP6, I-BABP or ILBP).

There are five FABP isoforms with relevant levels of expression in neural tissues: FABP3 (commonly known also as Heart-FABP), FABP5 (Epidermal-FABP), FABP7 (Brain-FABP), FABP8 (Myelin-FABP or PMP2), and FABP12 (Retinal-FABP). Each of them presents distinct spatio-temporal expression patterns (Owada et al., 1996; Liu et al., 2000). Many of them are significantly expressed in the brain, whereas FABP8 is apparently exclusive of PNS. Bellow there is a resume of the most prominent information available about each neural isoform.

#### FABP3

Significant expression of FABP3 is not detected in embryonic stages of rodent or in fetal human brains (Cheon et al., 2003; Saino-Saito et al., 2009). Perinatal FABP3 expression increases gradually, mainly confined to gray matter in rodents. In adult individuals, FABP3 is stably expressed in the neuronal layers of hippocampus, the cerebral neocortex, interneurons of the retina and the olfactory mitral cell layer, particularly in CA1 and CA2 portions (Owada, 2008; Saino-Saito et al., 2009). Its mRNA can also be detected in Purkinje and granulate cells of the cerebellum (Owada, 2008). ISH of mice brain slices localizes FABP3 expression in isocortex, cortex, pons and hippocampus (Figure 2C,G).

FABP3 shows somewhat preferential binding of ω-6 PUFAs, but affinities for all FAs range from 0.8 to 5 μM. FABP3 is thought to help to consolidate and maintain the differentiated status of neurons in adult brains through the utilization of PUFAs. Actually, brain ARA assimilation and its incorporation into phospholipids correlate with FABP3 expression levels, which is also necessary to maintain an optimal balance between ω-6 and ω-3 PUFAs in adult neurons (Murphy et al., 2005). FABP3 could also modulate dopamine signaling, since it is highly expressed in acetylcholinergic and terminals of glutamatergic neurons of dorsal *striatum*, and it can interact physically and modulate dopamine D2 receptors (D2R) in mice (Shioda et al., 2010). This was confirmed by dysfunctional response of D2R to amphetamines and to D2R-specific agonist Haloperidol in FABP3^-/-^ mice; whereas response to D1R-specific agonist SCH23390 was not impaired. The expression of FABP3 in dopaminergic neurons is still controversial, even between the same authors (Shioda et al., 2010, 2014). On the other hand, FABP3 can be found in the cochlea, as well as FABP7, not involved in sensing but present in different support cells, suggesting a non-redundant function of these proteins in modulation of the hearing process (Saino-Saito et al., 2010).

Regarding pathological conditions, FABP3 was found to be significantly decreased in frontal, occipital, and parietal cortices of patients suffering Down Syndrome; and it was also elevated in temporal cortex of patients with AD (Cheon et al., 2003; Sanchez-Font et al., 2003; Watanabe et al., 2007). FABP3 expression in dopaminergic neurons may promote MPTP and ARA-induced α-Synuclein aggregation (Shioda et al., 2014), the main component of the characteristic Lewy Bodies present in PD and related neurodegenerative diseases. Furthermore, aberrant expression of FABP3 may affect PUFA enrichment and alter membrane fluidity and signal transduction. Consequently, this deficiency could lead to cellular dysfunction in neurodegenerative disorders.

#### FABP5

FABP5 is the most ubiquitously expressed FABP. During midterm embryonic stages, it is particularly present in the ventricular germinal zone and the cerebral cortex, as well as in the stem cells differentiating into motor neurons or astrocytes. But its expression progressively decreases after birth until it is only weakly detected in the adult brain (Liu et al., 2010). This moderate expression is shared, with varying intensity in different brain regions, by neurons and glia (Figure 2D,G). Regarding mouse retina, FABP5 is found in the retinal ganglion cells, up to E14 strictly localized in the soma, but progressively migrating into axons of optic nerve by E18 and until P10 (Allen et al., 2001; Saino-Saito et al., 2009). This suggests that FABP5 may have a role in neurite outgrowth and axon development by supplying LCFA for phospholipid synthesis. FABP5 also shows high affinity for retinoic acid (RA) and, therefore, it is associated to neuronal survival and differentiation by activation of PPARβ/δ, not only through FAs but RA signaling as well. Actually, the latter depends on the distribution ratio between CRABP2 and FABP5, and it may be as well modulated by displacement from either protein by FAs, and particularly by DHA (Schug et al., 2007, 2008; Yu et al., 2012). Recent publications also link FABP5 to regulatory functions of estrogen receptors (Senga et al., 2018), which could be related to its heterodimerization to Retinoid X Receptors (RXRs), similarly to PPARs.

FABP5 expression is induced under pathological or stress conditions, for example, after axonal injury in peripheral nerves (De Leon et al., 1996), but not in Down Syndrome, AD or Bipolar Disorder (Cheon et al., 2003). Under oxidative stress conditions, FABP5 has been proved to work as a scavenging system of 4-Hydroxynonenal lipid peroxidation subproduct (Bennaars-Eiden et al., 2002). Altogether, we can summarize that FABP5 is expressed when the fate of the neural cell differentiation must be decided or when it has to adapt to stress or pathological conditions.

#### FABP7

In embryonic brains, FABP7 is highly expressed in the radial glia, in the ventricular and subventricular zones. After birth, the expression remains strongly positive in gray and white matter (Kurtz et al., 1994). In the neonatal brain, its expression is more evident in the olfactory nerve fiber layers, hippocampal dentate gyrus, and the cerebellar Purkinje cell layer. Finally, in adulthood, the expression decreases although it remains in the Schwann cells of olfactory nerve, in the radial glia of dentate gyrus, and in the glial cells located adjacent to the cerebellar Purkinje cells (Kurtz et al., 1994). Its mRNA expression in 8 weeks-old mice is evident only in olfactory bulb and the cerebellum (Figure 2E,G). FABP7 was proposed to be downstream regulator of Pax6 participating in the maintenance of pre-neurogenesis neuroepithelia, while its knock-down promotes neuronal differentiation (Arai et al., 2005). FABP7 and FABP3 are usually expressed in the same regions of the brain, with the later usually showing 10-time higher levels, although local concentration may vary (Pelsers et al., 2004). As mentioned before, both proteins are present in different support cells of the cochlea, suggesting a regulatory function of hearing signals (Saino-Saito et al., 2010). FABP7 is also expressed by glial progenitor cells located in the foregut and midgut during enteric nervous system development (Sasselli et al., 2012).

FABP7 preferentially binds ω-3 PUFAs and oleic acid (18:1n-9, OA) over ω-6 PUFAs, and shows lower affinity for SFAs (Xu et al., 1996; Balendiran et al., 2000; Hanhoff et al., 2002). Therefore, null mice display decreased DHA incorporation into PLs, with an increase in AA and PA instead (Owada, 2008). Its up-regulation during embryonic stages of development (and, probably, FABP5’s as well) is likely related to the proliferation and initial differentiation of neural stem cells and progenitorse, with an increasing demand of PUFAs, rather than to their maturation and integration (Liu et al., 2010). Actually, the fluorescent probe CDr3 was identified as a specific ligand for FABP7 in neural stem cells (Yun et al., 2012) and successfully employed for its flow cytometry isolation from both adult and embryonic mouse brains (Leong et al., 2013).

Regarding pathological conditions, FABP7 has been shown to be significantly increased in occipital cortex of patients with Down Syndrome, although no changes were detected in fetal brain (Cheon et al., 2003). Gene allele association studies showed correlation of FABP7 expression with schizophrenia and bipolar disorder (Saino-Saito et al., 2010).

#### FABP8

FABP8 can be abundantly and exclusively found, although unevenly distributed, in the myelin sheaths and Schwann cells of peripheral nerves, for example, in the human *nervus suralis*. It can represent up to 15% of total protein of bovine PNS myelin (Greenfield et al., 1973). In mice, mRNA levels increase gradually and the protein is detectable after P4 (Zenker et al., 2014). FABP8 is present only in minor amounts in CNS white matter, being more abundant in spinal cord and brain stem myelin. The expression levels of FABP8 vary both in intensity and distribution between different regions of a single nerve as well as between nerves (DeArmond et al., 1980).

FABP8 has a unique function in the organization and stabilization of myelin multilayers as it is capable of stacking phospholipid membranes synergistically with Myelin Basic Protein (Greenfield et al., 1973). Its higher expression during the early postnatal life also suggests a role in FAs uptake and lipid metabolism toward myelination of axons (Zenker et al., 2014). Notably, knock-out of one or both proteins does not affect myelin structure, and its only consequence is a minor retardation in motor nerve conduction (Zenker et al., 2014). However, several point mutations of FABP8 have been associated to the inherited neuropathy known as Charcot-Marie-Tooth disease (Motley et al., 2016; Punetha et al., 2018).

#### FABP12

The *FABP12* gene was the last one to be identified to code for a member of this family of proteins (Liu et al., 2008) and, hence, has not yet been thoroughly studied like other members. Its mRNA was found at high levels mainly in the retina and testis, and to a lesser extent in the cerebral cortex, kidney and epididymis of rat and mouse tissues (Smathers and Petersen, 2011). ISH of mouse brain sections shows a ubiquitous expression (Figure 2F,G). Under oxidative stress conditions, FABP12 protects retinal rod cells from peroxidation mediated by LCFA hydroperoxides (Guajardo et al., 2002).

### Nuclear Receptors-Transcription Factors

**FIGURE 3:**
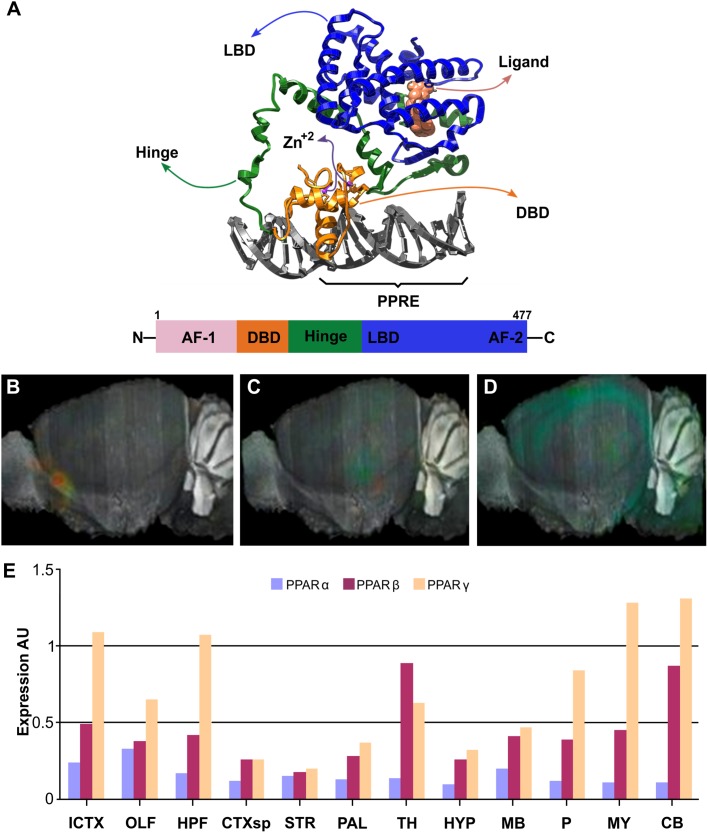
Structure and brain expression patterns of PPARs. **(A)** Cartoon based on crystal structure (PDB-ID: 3DZY) of the full-length PPARγ in a complex with RXRα and two accessory 13-amino acids peptides of Nuclear receptor coactivator 2 and a dsDNA containing the PPRE (Chandra et al., 2008). For clarity, only PPARγ, its synthetic agonist Rosiglitazone and the dsDNA are shown. N-terminal region of PPARγ is not visible because it was not determined due to high flexibility. **(B–D)** Brain left hemisphere sagittal projection showing expression of PPARα, PPARβ and PPARγ, respectively based on ISH data. **(E)** Quantification of relative brain expression from ISH data for neural FABPs isoforms. AU: Arbitrary units. For brain regions references see legend of Figure 1. Image credit for panels **B–D**: Allen Institute. © 2007 Allen Institute for Brain Science. Allen Mouse Brain Atlas. Available from: http://mouse.brain-map.org/search/. Panel **E** was constructed from data available through Allen Mouse Brain Atlas website.

In many tissues, FAs induce changes in gene expression through nuclear receptors of the PPARs family of transcription factors, ligand-activated nuclear receptors subfamily 1 group C (Capelli et al., 2016). PPARs structure consists of a variable N-terminal region, a conserved DNA binding domain, a variable hinge region, a conserved ligand binding domain, and a variable C-terminal region (Mangelsdorf et al., 1995). Heterodimerization of PPARs with Retinoid-X Receptor (RXR) depends on ligand and DNA binding, being affected also by posttranslational modifications (Berrabah et al., 2011; Anbalagan et al., 2012; Brunmeir and Xu, 2018). The DNA binding domain includes two segments with Zn-fingers motifs, which recognizes its target sequence or Response Element (RE). The N- and C-terminal regions include the Activation-Function 1 (AF1) and 2 (AF2), respectively, essential for interaction with coregulators and transcription modulation functions (Figure 3A). PPAR–RXR complexes recognize a direct repeat of the consensus sequence AGGTCA with a single nucleotide in between, also referred to as PPRE: AGGTCA-N_1_-AGGTCA. These features have been corroborated by the crystal structures available (PDB-IDs: 3E00, 3DZU, 3DZY) (Figure 3A) (Chandra et al., 2008). PPARs are thought to be permanently bound to PPREs as heterodimers with one of the three RXR isoforms (RXRα, RXRβ, or RXRγ). The lack of ligand bound to them promotes their repressor activity through association with corepressors, such as Nuclear Receptor Corepressor (NCoR) or the Silencing Mediator of Retinoid and Thyroid hormone receptor (SMART). Upon ligand binding, corepressors are released and co-activators recruited, including p300, CREB-binding protein, or Steroid Receptor Coactivator 1 (SRC1); promoting transcriptional activation of their target genes (Zoete et al., 2007).

PPARα was first identified as a new member of the steroid hormone receptor superfamily and proved to mediate the effects of hypolipidemic drugs commonly used in hyperlipidemias. These drugs were known as peroxisome proliferators, dubbing this receptor PPARs (Issemann and Green, 1990). Parallel identification of multiple isoforms in mammals and *Xenopus* sp. led to some controversy regarding their nomenclature, but nowadays it is accepted that three isoforms are present, comprising PPARα, PPARβ/δ, and PPARγ (Echeverria et al., 2016; Brunmeir and Xu, 2018). Besides FAs, PPARs have also been reported to be activated by other endogenous lipidic compounds, such as endocannabinoids (oleylethanolamide, arachidonylethanolamide, 2-arachidonyl-glycerol, etc.) and by RA (O’Sullivan, 2007; Schug et al., 2007). Unfortunately, little progress has been made identifying natural endogenous ligands which are specific or preferential for any of the PPARs isoforms due to their rather low affinity and similar specificities.

As mentioned above, the activation of PPARs modulates directly the expression of genes near the bound PPER. But a second mechanism of action has been proposed for certain isoforms, blocking or preventing the interaction of other transcription factors. The better studied example corresponds to the ligand-dependent SUMOylation of activated PPARγ that gets displaced from its PPRE and blocks NFκB interaction with its own response elements and, hence, limiting the inflammatory response (Ricote and Glass, 2007; Glass and Saijo, 2010). This mechanism is known as transrepression and may be a putative target for handling neuroinflmammation.

All PPARs isoforms were detected in the CNS employing multiple techniques, such as reverse transcription-quantitative PCR (RT-qPCR), immunohistochemistry (IHC), and ISH (Kainu et al., 1994; Braissant et al., 1996; Cullingford et al., 1998; Warden et al., 2016). However, despite the vast literature available, there is still some controversy regarding PPARs expression patterns. They are essential for embryonic and fetal development in mammals and, therefore, drug-inducible, tissue specific knockout models have been developed to study their specific roles, particularly for PPARγ null mice which is lethal (Gray et al., 2005; Wang et al., 2013; Rautureau et al., 2017). Double neuronal conditional knockout of PPARβ/δ and PPARγ exacerbates cytotoxicity of 1-methyl-4-phenyl-1,2,3,6-tetrahydropyridine (MPTP) on striatum dopaminergic neurons (Mounsey et al., 2015). Noteworthy, it is not rare to find co-expression of PPARs within a single cell. Some details are offered below about protein expression levels, together with mRNA and protein variants, and the connections of PPARs with brain pathophysiology.

#### PPARα

Human *PPARA* (or *NR1C1*) gene is located in chromosome 22q12-q13.1 and contains at least 9 exons, with PPARα encoded as a 468 amino acid protein (52.23 kDa). A shorter variant is also described by alternative splicing that introduces an early stop codon after the DNA binding domain and, therefore, is not ligand-sensitive. PPARα shows a wide and similar expression in all tissues, with more prominent levels in liver, intestine, kidney, heart, and skeletal muscle; and its presence is also reported in brown adipose tissue. The higher expression levels of PPARα in these tissues correlate with their high capacities for FAs oxidation, and its main function seems to be related to the control of energy expenditure through lipid catabolism and the adaptation to different nutritional states, such as the postprandial period or fasting. Regarding CNS expression, the GTEx project detected similar levels of *PPARA* mRNA in caudate, pituitary gland, hypothalamus, hippocampus, cerebral cortex, and cerebellum. Furthermore, ISH of brain section showed very similar expression throughout regions and weaker than other isoforms (Figure 3B,E). Targeted expression analysis of PPARα reported it in olfactory bulb, retina, cerebellum, and hippocampus (Braissant et al., 1996; Moreno et al., 2004; Rivera et al., 2014). PPARα has been localized in neurons (Cristiano et al., 2001; Santos et al., 2005), oligodendrocytes (Kainu et al., 1994), microglia (Warden et al., 2016), and specially in astrocytes (Cristiano et al., 2001; Chistyakov et al., 2015).

PPARα shows relatively higher affinities for FAs compared to alternative endogenous agonists (eicosanoids and endocannabinoids), and it can be activated also by hypolimidemic drugs, but generally not or only weakly by non-steroidal anti-inflammatory drugs (NSAIDs) and anti-diabetic thiazolidinediones (Corton et al., 2000). PPARα functions in the CNS are still unclear. Its proposed functions include the regulation of sleep process (Murillo-Rodriguez, 2017), also have an impact on learning and memory consolidation. Nevertheless, it has been shown that PPAR*α* agonists such as Wy-14643 reduce A*β*-derived oxidative damage by increasing catalase activity, and activate the Wnt/*β*-Catenin survival pathway (Santos et al., 2005). Furthermore, PPARα activation by statins is responsible for boosting BDNF production, that mediates cognitive improvement in another mouse mode of AD (Roy and Pahan, 2015).

#### PPARβ/δ

Gene structure of *PPARD* (*NR1C2*) gene in chromosome 6p21.2-21.1 consists of 11 exons, with the ORF spanning from exon 4 to exon 9 and coding for a 441 amino acids protein termed PPARδ1 (49,09 kDa). Alternative splicing gives rise to shorter isoforms: 80 amino acids shorter isoform 2 (PPARδ2) is the product of an early stop signal due to overtranscription of the 3’-end of exon 8 that introduces an in-frame non-sense codon; variant 4 lacks 98 amino acids (44 to 141) due to exon skipping; while variant 3 is the result of an alternative transcription initiation site with three new N-terminal amino acids instead of the first 43 residues of PPARδ1 (coded by exon 4). Another four alternative transcription initiation sites may be active in *PPARD* downstream to exon 1 and associated to alternative exon 2 (2a′, 2c, 2c′ and 2e) (Lundell et al., 2007). These mRNA 5′-UTR variants do not alter the final ORF but probably are related to translational regulation of PPARβ/δ. The different isoforms are suspected to show distinctive spatio-temporal expression patterns and/or specific functions. For example, PPARδ2 has been proposed to act as a dominant negative form of PPARδ1 (Dickey et al., 2016).

*PPARD* expression is broad and is particularly high in tissues associated with FAs metabolism, such as the gastrointestinal tract, heart, kidney, skeletal muscle, fat, and skin. Its systemic physiological function is to coordinate and balance the usage levels of FAs and glucose in muscle and liver. However, PPARβ/δ is also the most predominant PPARs isoform in the human CNS and has a ubiquitous expression in the rat brain (Figure 3C,E). Despite, all PPARs are co-expressed in developing CNS, PPARβ/δ remains with high expression levels in adult rats (Braissant et al., 1996), suggesting a role in brain development, mielynation, and neuronal function. It was localized in olfactory bulb, cerebral cortex, basal ganglia, hippocampus, hypothalamus, cerebellum, and spinal cord, among others brain areas (Xing et al., 1995; Braissant et al., 1996; Moreno et al., 2004). In mouse models, PPARβ/δ is widely distributed and has been found in neurons of the prefrontal cortex, nucleus accumbens, amydala, cerebellum, hypothalamus, and spinal cord, at mRNA and protein expression levels (Woods et al., 2003; Warden et al., 2016). Regarding cell types, PPARβ/δ is mainly expressed in neurons (Hall et al., 2008), but it has also been found in oligondendrocytes of the corpus callosum (Moreno et al., 2004), and in primary cultures of cortical and cerebral astrocytes (Cristiano et al., 2001). Among the glial cells, PPARβ/δ only co-localized with oligondendrocytes of the corpus callosum (Woods et al., 2003), but no with astrocytes and microglia. *In vitro*, agonists of PPARβ/δ induce the differentiation of SH-SY5Y cells (Di Loreto et al., 2007).

PPARβ/δ shows preferentially affinities for PUFAs, compared to other FAs and eicosanoids, and is selectively activated by bezafibrate (hypolipidemic drug), GW2433 and L-65041 (NSAIDs) among synthetic agonists (Corton et al., 2000). Regarding its pathological links, PPARβ/δ is repressed in patients with Huntington Disease (HD) and its pharmacological activation improves motor function, reduces neurodegeneration, and increases neuronal survival in a HD mice and cellular models (Dickey et al., 2016). The novel PPARβ/δ agonist gemfibrozil is believed to promote oligodentrocyte differentiation by increasing the expression of genes required for myelin formation (Jana et al., 2012) and could potentially be employed against demyelination-related pathologies.

#### PPARγ

*PPARG* (*NR1C3*) gene is located in chromosome 3p25. At least four transcriptional start sites actively transcribe different mRNAs with functional cDNAs coding for PPARγ isoforms. While variants 1, 3 and 4 code for the same PPARγ1 isoform (477 amino acids, 54.68 kDa), mRNA variant 2 codes for PPARγ2 which has an additional 28 amino acids at the N-terminus (505 amino acids, 57,62 kDA). PPARγ1 is expressed in a broad variety of cells including immune and brain cells, whereas PPARγ2 is highly abundant in adipose tissue and is considered the master regulator of adipocyte differentiation, where it controls FAs uptake and storage in lipid droplets as triglycerides. Within the CNS, PPARγ was found to show a more discrete pattern of expression than PPARβ/δ, and to be slightly enriched in the hippocampus, being identified both in neurons and glial cells, including microglia, (Bernardo et al., 2000; Roth et al., 2003; Heneka and Landreth, 2007; Sarruf et al., 2009; Warden et al., 2016; Villapol, 2018). Expression analysis in mice brain by ISH showed hippocampus, isocortex, cerebellum and medulla higher levels of *PPARG* mRNAs (Figure 3D,E). In the neuroblastome cell line SH-SY5Y, the activation of PPARγ by synthetic agonists promotes neurite outgrowth and neuronal differentiation (Miglio et al., 2009).

PPARγ shows higher affinities for PUFAs than for MUFAs, and it cannot be activated by SFAs, and its response to eicosanoids, hipolipidemic and NSAIDs is diverse, but it is generally activated by thiazolidinediones (Corton et al., 2000). PPARγ is probably the most studied isoform regarding its functions in immune cells, and its interest in neuropathological processes points toward its strong anti-inflammatory effects (Corona and Duchen, 2015). PPARγ activation can induce differentiation of oligodendrocytes and protects them from TNFα toxicity (Bernardo et al., 2009, 2017; De Nuccio et al., 2015). Furthermore, natural and synthetic agonists PPARγ can control brain inflammation processes by shutting-down proinflammatory phenotype of activated microglia, and inhibiting the expression of surface antigens or the synthesis of proinflammatory signals, such as prostaglandins and nitric oxide (Bernardo and Minghetti, 2006). PPARγ can be either positively or negatively modulated by phosphorylation, according to the residue that is modified (Shao et al., 1998; Anbalagan et al., 2012; Brunmeir and Xu, 2018), and can be sent for degradation via the Ubiquitine-Proteasome pathway. PPARγ ability to transrepress NFκB inhibits, or at least limits, the inflammatory response, a regulatory mechanism activated by SUMOylation of its N-terminal region (Ricote and Glass, 2007; Diezko and Suske, 2013; Glass and Saijo, 2010). This could be a putative target for minimizing the neuroinflammatory condition characteristic of many neurodegenerative diseases, such as PAD and AD. Actually, multiple drugs designed as agonist of PPARγ have been and tested in AD models showed to reduce the β-amyloid accumulation, its cytotoxicity and stimulation of inflammatory cytokines (Combs et al., 2000; Sastre et al., 2006; Zolezzi and Inestrosa, 2013; Bonet-Costa et al., 2016).

## Future Perspectives

**FIGURE 4:**
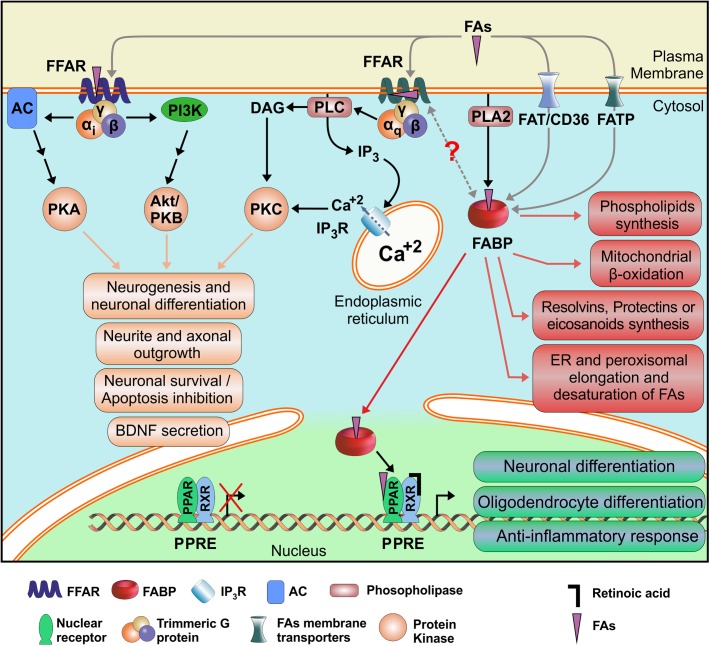
Schematic mechanism proposed for integration of the FAs Receptors System. Alternative FAs receptors are shown along with their demonstrated or putative interactions and their better characterized cellular effects. FFAR: Free Fatty Acid Receptor; FABP: Fatty Acid Binding Protein; PPAR: Peroxisome Proliferator-Activated Receptor; RXR: Retinoid X Receptor; FA: Fatty Acid; PPRE: PPAR Response Element. AC: Adenylate Cyclase; DAG: Diacylglycerol; FATP: FAT/CD36: Fatty Acid Translocase; Fatty Acid Transport Protein; IP3: Inositol (1,4,5)-Trisphosphate; IP_3_R: Inositol trisphosphate Receptor; PI3K: Phosphatidyl Inositol (3,4,5)-Trisphosphate Kinase; PLA2: Phospholipase A2; PLC: Phospholipase C; PKA: Protein Kinase A; PKAB/Akt: Protein Kinase B; PKC: Protein Kinase C.

Fatty acids participate in essential and diverse cellular processes involving neurons and glial cells, ranging from embryonic and perinatal development of tissues, including the CNS, to cognitive functions such as memory and learning. Therefore, they are unavoidably involved in neuropathological processes, including traumatic brain injury and neurodegenerative diseases. But, due to its rather simple chemical structures and low solubility, FAs require specific proteins that could recognize them and mediate its regulatory or signaling functions. Membrane receptors FFARs, cytosolic transport proteins FABPs and nuclear transcription factors PPARs are the preferential mediators of FAs functions. Taken together, and considering that almost all cell types express at least one member of each of these three families of proteins, we could hypothesize that they may be working as a complex, but coordinated sensory system for FAs. Actually, functional interaction of FABPs and PPARs has already been proved in hepatocytes and adipocytes (Smith et al., 2008; Hostetler et al., 2009). The identification of two possible ligand binding sites in FFAR1 structures in opposite sides of the membrane opens the possibility that they could be regulated and/or activated by intracellular lipids as well as by external lipokines, for example released by PLA_2_ enzymes, but also by interaction with FABPs. FABP-FFAR interaction, though not yet proved, could be understood either by unloading FAs cargo from the FABPs to activate the FFARs, or as a termination mechanism of FFAR signaling by retrieval of ligands by the FABPs from the FFARs. This is an interesting possibility that would integrate all FAs receptors (Figure 4), and its better understanding would definitely help in the design of new drugs with increased specificity and selectivity for its primary targets, avoiding undesired adverse effects.

Fatty acids signaling effects are usually mixed with those displayed by their multiple derivatives, adding a new layer of complexity to distinguish direct cellular responses free FAs signaling. Although specific receptors have been identified for endocannabinoids, eicosanoids, resolvins, protectines, lysophasphatidic acids, monoacylglycerols, N-acylethanolamines, and so on, crosstalk between the different receptors should not be neglected or discarded. Cellular responses are the result of the integration of a myriad of external and internal signals, as evidenced by the convergence of signaling pathways. Particularly, a functional interaction between FFAR2 and FFAR3 to form an heteromeric receptor has been proposed in primary monocytes and macrophages, and during heterologous expression in HEK293 cells (Ang et al., 2018). Considering that the origin of the short chain carboxylic acids available in mammals’ bloodstream is exclusive from the microbiota colonizing their body, FFAR2 and FFAR3 may act as a remote sensory system to prevent infections, activating the proliferation and modulating the reactivity of immune cells in preparation against a growing focus of bacteria (Ulven, 2012).

Signaling of PUFAs is intimately related to RA signaling, as mentioned above by the similar activation of FFAR1. Furthermore, and similarly to what happens with FABP5 and CRABP2, PPARγ has been described to be activated by RA, as well as RXRs by DHA. In this case, FABP5 translocate its ligand, either DHA or RA, to the nucleus and unload it to the PPARβ/δ (Noy, 2016). Similarly, CRABP2 would do the same to RXR. Therefore, FABP/CRABP2 biological interaction with PPAR/RXXR is not only direct but they also compete for the same ligands, increasing the possibilities of diverse responses mounted and the subset of target genes affected. Regarding this, one must remember that many nuclear receptors heterodimerize with RXRs and, therefore the final cellular response observed is also affected by their presence and ligands (Zhang et al., 2015).

Another utility for FABPs is their detection as early biomarkers of tissue injuries, and increasing evidence is being recorded for the early or differential diagnosis of diverse pathologies (Pelsers et al., 2004, 2005; Wunderlich et al., 2005). Their main advantages are related to their fast release, as close as 30 min to the injurious event, and their relatively fast clearance. Consequently, FABPs presence in plasma or cephaloraquidean liquid reports for recent trauma, allowing for the differentiation of consecutive events separated by less than 24 h. Neural FABPs detection has been correlated with traumatic brain injury and certain gliomas overexpressing FABP5 or FABP7 (De Rosa et al., 2012; Walder et al., 2013).

Finally, the increasing complexity of the lipid-sensing system available in humans raises the question if the regulatory and signaling functions of FAs can be individually discriminated and specifically targeted for the treatment of pathological conditions. Significant effort is being invested in the development of new drugs that would allow for their precise manipulation and the better treatment of brain injuries, neurological or neurodegenerative diseases by taking advantage of the neuroprotective and anti-inflammatory properties, particularly of PUFAs, employing either FFARs, FABPs, or PPARs as drug targets (Holliday et al., 2012; Wang et al., 2016; Zhou et al., 2016; Li et al., 2018a,b,c, 2019; Shang et al., 2018). Specificity will come together with our better understanding of how these proteins work. For example, an important step forward in this direction is the recent variation analysis of the PPRE sequence for PPARα/RXRα complex that resulted in the ideal sequence WAWVT-RGGBBA-H-RGKTYA (where W = A or T; V = not T; R = A or G; B = not A; H = not G; K = G or T; Y = T or C) as an optimized DNA sequence for PPARα (underlined) and RXRα binding (not underlined) (Tzeng et al., 2015). Noteworthy, although stronger DNA binding of RXRα to PPRE led to higher transcription rates, this is not always the case for PPARα. New candidate target genes have been identified employing this optimized sequence to screen genomic databases as being regulated by PPARα, and that could not be recognized by the consensus sequence for PPRE, improving our understanding of PPARs isoform specific functions. This could also help to development of drugs that could selectively affect only one PPAR isoform and/or only its functions regarding a certain subset of genes under its control.

Another elements that must be considered are non-coding microRNAs (miRs), which act usually as negative regulators of gene expression in the CNS, and several of them have been reported to participate in the regulation exhibited by lipids (Wnuk and Kajta, 2017). For example, the expression levels of miR-21 are decreased by DHA treatment of SH-SY5Y neuroblastome cells, showing an inverse correlation with PPARα levels (Fu et al., 2017). The miR-21 is thought to destabilize PPARα mRNAs and reduce its translation (Chen et al., 2017). Treatment with DHA plus salicylic acid promotes PPARα-RXRα heterodimer formation that correlates with a reduction of miR-21. Together, they promote PSD-95, BDNF and GDNF neuronal differentiation markers and reduce NFκB, COX-2, caspase 3 levels proinflammatory markers (Fu et al., 2017). Similarly, miR-499a negatively regulates the level of expression of PPARγ in microglia cells, promoting the expression of proinflammatory markers, such as iba-1, TNFα and IL-1β (Yu et al., 2018). Finally, PPARs can be activated by valproic acid and its short/medium-chain derivatives, a long time used family of anticonvulsants to treat epilepsy, that has also showed beneficial effects against autism, maniac and bipolar disorders (Lampen et al., 2001; Hunsberger et al., 2013; Hirsch et al., 2018). Its beneficial effects as a promoter of neuroregeneration, neurodifferentiation and neuroprotection (Liu et al., 2012; Foti et al., 2013; Oikawa and Sng, 2016) may come from the modulation of the expression of multiple miRs through PPARs, either by their transrepression or transactivation (Dharap et al., 2015). FFARs may also participate of miRs expression, such as miR-143 that is controlled in adipocytes by both PPARγ and FFAR4 activation (Bae et al., 2017). These line of evidence has recently pushed miRs to be evaluated as novel therapeutic targets against neurodegenerative diseases, such as PD o AD.

Drug discovery strategies focused on FFARs, FABPs and PPARs have already started aiming at neurological and also other pathologies like diabetes, obesity, leukemia, cancer, and so on (Kaczocha et al., 2014; Nikaido et al., 2015; Corona and Duchen, 2016; Wang et al., 2016; Cheng et al., 2019), paving the way for new developments and facilitating drug repurposing, what may save years of research and development. Noteworthy, in the case of FFARs with multiple signal transduction pathways, the concept of biased signaling is of particular interest, where a partial agonistic ligand may only and selectively activate a single pathway over the others available for that receptor. For example, FFARs partial agonist could activate β-Arrestin-mediated but not G protein-mediated signaling (Mancini et al., 2015). This kind of developments has attracted substantial attention for its potential to model molecular differences in receptors functionality that could yield enhanced therapeutic strategies, with improved efficacy and reduced adverse effects (Whalen et al., 2011; Kenakin and Christopoulos, 2013; Correll and McKittrick, 2014).

## Author Contributions

All authors listed have made a substantial, direct and intellectual contribution to the work, and approved it for publication.

## Conflict of Interest Statement

The authors declare that the research was conducted in the absence of any commercial or financial relationships that could be construed as a potential conflict of interest.

## Footnotes

1 www.proteinatlas.org (accessed January 3, 2019)

2 http://portal.brain-map.org (accessed January 3, 2019)
